# Bilateral subthalamic nucleus deep brain stimulation for refractory isolated cervical dystonia

**DOI:** 10.1038/s41598-022-11841-1

**Published:** 2022-05-10

**Authors:** Feng Yin, Mingming Zhao, Xin Yan, Tong Li, Hui Chen, Jianguang Li, Shouming Cao, Hui Guo, Shuang Liu

**Affiliations:** Department of Neurosurgery, Aerospace Center Hospital, No. 15, Yuquan Road, Haidian District, Beijing, China

**Keywords:** Neuroscience, Neurology

## Abstract

Subthalamic nucleus (STN) deep brain stimulation (DBS) has been proven to be an alternative target choice for refractory isolated cervical dystonia (CD). However, assessments of its short and long-term safety, efficacy, and sustained effectiveness have been limited to few reports. Here, we evaluated nine consecutive refractory isolated CD patients who underwent bilateral STN DBS and accepted to short and long-term follow-up in this retrospective study. Seven time points were used to see the Toronto Western Spasmodic Torticollis Rating Scale (TWSTRS) scores (pre-operation [baseline], 1, 3, 6, 12, 24 months post-operation and last follow-up) to assess improvement of dystonic symptoms. The 36-item Short-Form General Health Survey (SF-36) scores obtained at pre-operation and last follow-up to assess the changes in quality of life. All patients tolerated surgery well and acquired observable clinical benefits from STN DBS therapy. All patients achieved a considerable improvement in quality of life at the last follow-up. The hardware-related adverse events can be tolerated and the stimulation-related adverse events can be ameliorated by programming. Our data support the idea that bilateral STN DBS is a safety and effective method for the treatment of refractory isolated CD, with persistent and remarkable improvement in both movement and quality of life.

## Introduction

Cervical dystonia (CD) is a focal dystonia characterized by sustained or intermittent involuntary contractions of the neck muscles causing turns or tilts in jerky movements or awkward postures of the head^1^. CD is the most common form of focal dystonia, with an approximated prevalence of 28–183 per million people^2^. The complex interaction of motor function impairment, neck pain, and disgrace of CD patients lead to the quality of life reduced seriously due to significant restrictions in daily activities and social participation^3–5^.

The conventional treatments of CD, which include of oral medication, botulinum toxin injections and physical therapy, are indefinite, causing adverse effects or unsustained effect^6^. Patients have no alternative but to surgical intervention when show resistance to conventional treatment^2,7^. In spite of selective peripheral denervation (SPD) usually gives a satisfactory result in most of the patients, dystonia may recur because of either regeneration of the denervated muscles or disease progression to muscles in the cervical region that were not denervated beforehand^7^. Deep brain stimulation (DBS) has been widely used in the treatment of CD and internal globus pallidus (GPi) is the most prevalently used target, its safety and efficacy have been validated^8^, but stimulation-induced side effects (such as bradykinesia and axial motor signs) are frequent and insurmountable^9^. Its high-energy consumption is another drawback^10^. The effectiveness of subthalamic nucleus (STN) stimulation has shown to be an alternative target choice in the treatment for isolated CD, but only displayed in a few of studies^11,12^.

In this retrospective study, we explore the effect of bilateral STN DBS on safety, efficacy, sustainability and quality of life in a series of patients with refractory isolated CD.

## Results

Patient demographics are shown in Table 1. Nine patients (3 men and 6 women) were enrolled in the study. The mean age of onset and duration of disease were 40.9 ± 12.3 (18–62) years and 4.1 ± 2.5 (range 1.5–9) years, respectively. The average age at surgery was 44.9 ± 11.9 (range 27–69) years, and the mean follow-up time was 46.6 ± 15.8 (range 24–68) months (Table 1). All patients received bilateral STN DBS and complete the 24 months visit. All patients had prominent CD. Some dystonia was also present in other body regions in 3 patients, but the symptoms were mild. Only one patient received battery replacement 42 months after DBS due to battery depletion. Before DBS, the average TWSTRS severity, disability, and pain subscores and total scores were 22.4 ± 3.2 (range 19.0–28.0), 19.3 ± 3.9 (range 13.0–25.0), 6.1 ± 4.4 (range 0–12.0), and 47.9 ± 9.5 (range 32.0–60.0), respectively (Tables 2 and 3).

**Table 1:** Clinical characteristics and DBS devices model for each patient with isolated cervical dystonia. *DBS* deep brain stimulation, *F* female, *M* male, *SPD* selective peripheral denervation, *TCM* traditional Chinese medicine, *SD* standard deviation, *yrs* years, *mos* months.

Patient No	Sex	Age at onset (yrs)	Duration of disease (yrs)	Site of onset	Baseline distribution of dystonia	Type of dystonic movement	Age at DBS surgery (yrs)	Duration of follow-up (mos)	Treatment before DBS	DBS lead model	Neurostimulator model
1	F	42	1.5	Cervical	Neck	Tonic	43	68	Carbamazepine, TCM	L301 (PINS)	G101A
2	F	30	3	Cervical	Neck	Phasic	33	60	TCM, botulinum toxin, SPD	L301 (PINS)	G102
3	M	42	3	Cervical	Neck	Tonic	45	60	Baclofen, botulinum toxin	L301 (PINS)	G102
4	F	45	2	Cervical	Neck	Phasic	47	54	TCM, baclofen	3389S (Medtronic)	RC
5	M	40	3	Cervical	Neck/Eyes/Mouth	Phasic	43	51	Tiapride, haloperidol, alprazolam, clonazepam, baclofen	L301 (PINS)	G102R
6	F	18	9	Cervical	Neck/Arm/Foot	Phasic	27	43	TCM, botulinum toxin, clonazepam, quetiapine, l-levodopa, SPD	L301 (PINS)	G102R
7	M	51	3	Eyes	Neck/Eyes/Mouth	Tonic	54	30	Clonazepam, baclofen	L301 (PINS)	G102R
8	F	38	5	Cervical	Neck	Phasic	43	29	Clonazepam, trihexyphenidyl, botulinum toxin	L301 (PINS)	G102
9	F	62	7	Cervical	Neck	Tonic	69	24	Baclofen, trihexyphenidyl	L301 (PINS)	G102RZ
Mean ± SD		40.9 ± 12.3	4.1 ± 2.5				44.9 ± 11.9	46.6 ± 15.8

**Table 2:** TWSTRS scores at baseline and after bilateral STN DBS. *TWSTRS* Toronto Western Spasmodic Torticollis Rating Scale, *STN* subthalamic nucleus, *DBS* deep brain stimulation.

Patient no.	TWSTRS (severity/disability/pain subscale scores)							Stimulation parameters at last follow-up
	Baseline	Month 1	Month 3	Month 6	Month 12	Month 24	Last follow-up	
1	39 (20/16/3)	30.5 (16/12/2.5)	14 (6/7/1)	3 (2/1/0)	0 (0/0/0)	0 (0/0/0)	0 (0/0/0)	Left:1.0 V/130 Hz/60us case (+) 6 (−) Right:1.0 V/130 Hz/60us case (+) 2 (−)
2	57 (26/22/9)	36 (17/14/5)	10 (3/5/2)	0 (0/0/0)	0 (0/0/0)	0 (0/0/0)	0 (0/0/0)	Left:2.5 V/140 Hz/60us case (+) 5 (−) Right:2.5 V/140 Hz/60us case (+) 1 (−)
3	51 (24/20/7)	43 (19/18/6)	31 (13/15/3)	25 (10/12/3)	15 (7/6/2)	9 (5/2/2)	0 (0/0/0)	Left:2.9 V/132 Hz/60us case (+) 7 (−) Right:2.5 V/132 Hz/70us case (+) 1 (−)
4	41 (20/15/6)	36 (18/13/5)	9 (6/3/0)	0 (0/0/0)	0 (0/0/0)	0 (0/0/0)	0 (0/0/0)	Left:2.7 V/150 Hz/60us case (+) 7 (−) Right:2.8 V/150 Hz/60us case (+) 3 (−)
5	60 (28/25/7)	51 (22/23/6)	50 (20/24/6)	52 (22/24/6)	48 (21/22/5)	54 (26/23/5)	15 (7/5/3)	Left:2.4 mA/130 Hz/60us case (+) 5 (−) Right:2.4 mA/130 Hz/60us case (+) 4 (−)
6	43 (20/23/0)	38 (18/20/0)	20 (10/10/0)	0 (0/0/0)	0 (0/0/0)	0 (0/0/0)	0 (0/0/0)	Left:1.6 V/150 Hz/60us case (+) 7 (−) Right:3.1 V/150 Hz/60us case (+) 2 (−)
7	56 (24/20/12)	35 (16/12/7)	30 (13/10/7)	25 (11/9/5)	0 (0/0/0)	0 (0/0/0)	0 (0/0/0)	Left:2.9 V/130 Hz/60us case (+) 6 (−) Right:2.05 V/130 Hz/60us case (+) 4 (−)
8	32 (19/13/0)	20 (12/8/0)	11 (8/3/0)	4 (3/1/0)	0 (0/0/0)	0 (0/0/0)	0 (0/0/0)	Left:2.3 V/130 Hz/60us case (+) 6 (−) Right:2.3 V/130 Hz/60us case (+) 2 (−)
9	52 (21/20/11)	46 (19/19/8)	25 (12/10/3)	16 (8/5/3)	3 (2/1/0)	3 (2/1/0)	3 (2/1/0)	Left:2.5 V/130 Hz/60us case (+) 8 (−) Right:2.5 V/130 Hz/60us case (+) 3 (−)

**Table 3:** The outcomes for TWSTRS and improvement from baseline. *TWSTRS* Toronto Western Spasmodic Torticollis Rating Scale. Mean values are expressed ± SD.

TWSTRS	Baseline	Month 1	Month 3	Month 6	Month 12	Month 24	Last follow-up
**Total score**	47.9 ± 9.5	37.3 ± 9.0	22.2 ± 13.4	13.9 ± 17.7	7.3 ± 16.0	7.3 ± 17.8	2.0 ± 5.0
Mean % change from baseline		22.2 ± 11.7	55.3 ± 20.3	74.3 ± 29.8	87.2 ± 27.0	87.3 ± 29.6	96.6 ± 8.3
*p* value*		0.001	< 0.001	0.008	0.008	0.008	0.008
**Severity score**	22.4 ± 3.2	17.4 ± 2.7	10.1 ± 5.1	6.2 ± 7.4	3.3 ± 7.0	3.7 ± 8.5	1.0 ± 2.3
Mean % change from baseline		21.8 ± 10.9	55.5 ± 18.1	74.4 ± 27.2	87.4 ± 25.3	86.2 ± 30.5	96.2 ± 8.5
*p* value*		0.001	< 0.001	< 0.001	0.007	0.007	0.007
**Disability score**	19.3 ± 3.9	15.4 ± 4.8	9.7 ± 6.6	5.8 ± 8.1	3.2 ± 7.3	2.9 ± 7.6	0.7 ± 1.7
Mean % change from baseline		21.0 ± 14.1	52.9 ± 25.3	73.3 ± 33.9	86.3 ± 29.6	88.1 ± 30.2	97.2 ± 6.7
*p* value		0.002	< 0.001	0.007	0.008	0.008	0.007
Pain score	6.1 ± 4.4	4.4 ± 2.9	2.4 ± 2.6	1.9 ± 2.4	0.8 ± 1.7	0.8 ± 1.7	0.3 ± 1.0

*p values for improvement comparisons between baseline and follow-up time points after operation, as assessed using two tailed paired-sample *t* tests or Wilcoxon matched-pairs signed-rank tests.

### Clinical outcomes

**Figure 1:**
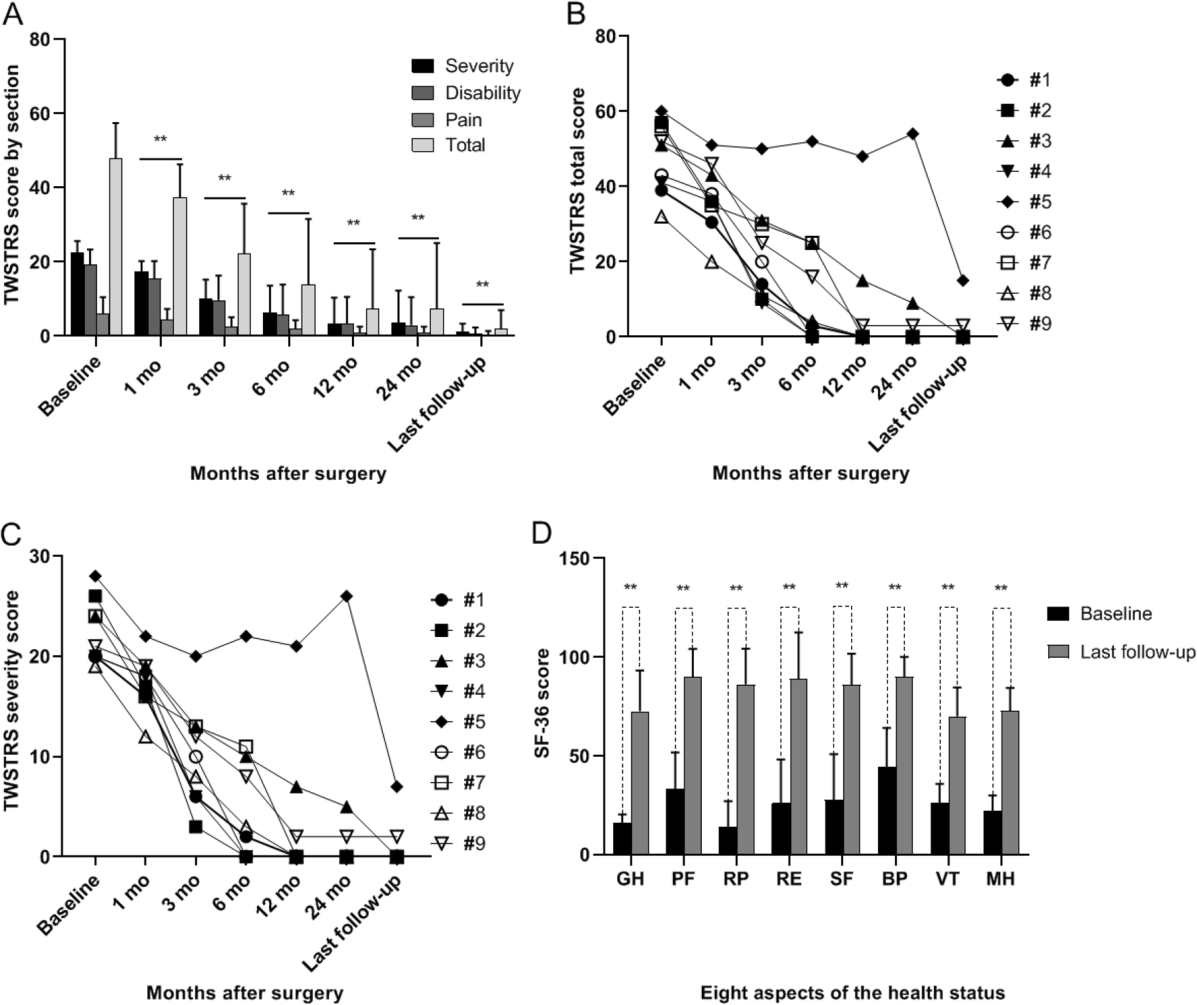
Mean score for TWSTRS severity, disability, pain, and total scores at various time points before (baseline) and after operation (**A**). Line graphs show individual scores of the TWSTRS total score (**B**) and severity score (**C**) at various time points before (baseline) and after operation. Mean score for the baseline and the last follow-up assessed by the 36-item Short General Health Survey (**D**). *GH* general health, *PF* physical functioning, *RP* physical-role functioning, *RE* role-emotional functioning, *SF* social functioning, *BP* bodily pain, *VT* vitality, *MH* mental health. **p < 0.01.

Effects of DBS on TWSTRS scores are detailed in the Table 2. Compared to baseline scores, the mean improvements in the TWSTRS severity scores were 21.8% ± 10.9% (range 9.5–36.8%; p = 0.001) at 1 month postsurgery, 55.5% ± 18.1% (range 28.6–88.5%; p < 0.001) at 3 months postsurgery, 74.4% ± 27.2% (range 21.4–100%; p < 0.001) at 6 months postsurgery, 87.4% ± 25.3% (range 25.0–100%; p = 0.007) at 12 months postsurgery, 86.2% ± 30.5% (range 7.1–100%; p = 0.007) at 24 months postsurgery, and 96.2% ± 8.5% (range 75.0–100%; p = 0.007) at the last follow-up. Similar improvements were seen in the TWSTRS total scores, with 22.2% ± 11.7% (range 11.5–37.5%; p = 0.001) at 1 month postsurgery, 55.3% ± 20.3% (range 16.7–82.5%; p < 0.001) at 3 months postsurgery, 74.3% ± 29.8% (range 13.3–100%; p = 0.008) at 6 months postsurgery, 87.2% ± 27.0% (range 20.0–100%; p = 0.008) at 12 months postsurgery, 87.3% ± 29.6% (range 10–100%; p = 0.008) at 24 months postsurgery, and 96.6% ± 8.3% (range 75.0–100%; p = 0.008) at the last follow-up (Table 3 and Fig. 1a).

Compared to baseline scores, the mean improvements in the TWSTRS disability scores were 21.0 ± 14.1 (range 5.0–40.0%; p = 0.002) at 1 month postsurgery, 52.9 ± 25.3 (range 4.0–80.0%; p < 0.001) at 3 months postsurgery, 73.3 ± 33.9 (range 4.0–100%; p = 0.007) at 6 months postsurgery, 86.3 ± 29.6 (range 12.0–100%; p = 0.008) at 12 months postsurgery, 88.1 ± 30.2 (range 8.0–100%; p = 0.008) at 24 months postsurgery, and 97.2 ± 6.7 (range 80.0–100%; p = 0.007) at the last follow-up (Table 3 and Fig. 1a). We also observed significant improvements postoperatively compared to baseline in the TWSTRS pain scores (Table 3).

Patient-5 is a special case. He presented with sustained cervical dystonia before DBS, but changed to paroxysmal episodes under constant-voltage stimulation after DBS. One day, the patient had a very severe episode, and we tried constant-current stimulation by remote programming, and the episode stopped immediately. After that, the symptoms gradually improved and relatively satisfactory results were obtained at the last follow up (Fig. 1b,c).

The quality of life was also improved significantly for all the patients at the last follow-up. Changes in SF-36 scores after chronic stimulation reflect this improvement (Table 4 and Fig. 1d).

**Table 4:** The outcomes for 36-item Short-Form General Health Survey. *SF-36* The 36-item Short-Form General Health Survey, *SD* standard deviation.

SF-36 subscale	Score (mean ± SD)		*p* value
	Baseline	Last follow-up	
General health	16.1 ± 4.2	72.6 ± 20.7	0.008
Physical functioning	33.3 ± 18.4	90.0 ± 14.1	0.007
Role physical	13.9 ± 13.2	86.1 ± 18.2	0.006
Role emotional	25.9 ± 22.2	88.9 ± 23.6	< 0.001
Social functional	27.7 ± 23.1	85.9 ± 15.8	0.008
Body pain	44.2 ± 19.9	90.0 ± 10.0	< 0.001
Vitality	26.1 ± 9.6	70.0 ± 14.6	0.008
Mental health	22.2 ± 7.8	72.9 ± 11.5	0.007

### Predictors for movement improvement

We assessed correlations between the final TWSTRS severity improvement and baseline factors, as well as duration of stimulation. However, there were no significant predictors for long-term movement improvement (sex, p = 0.714; type of dystonic movement, p = 1.0; age at disease onset, p = 0.513; duration of disease, p = 0.558; age at surgery, p = 0.507; duration of stimulation, p = 0.332; baseline TWSTRS severity, p = 0.206).

### Stimulation parameters

The stimulation parameters were shown in Table 2. Post-operative imaging confirmed all of electrodes were implanted near the predetermined location (central in the dorsal STN). A total of 18 DBS electrodes were placed in 9 patients and all used monopolar stimulus mode. At the last follow-up visit, the mean pulse width was 60.7 ± 2.3 us, the mean frequency was 134.3 ± 6.5 Hz, 8 patients using constant-voltage stimulation (mean amplitude was 2.3 ± 0.6 V) and one patient using constant-current stimulation (bilateral 2.4 mA). At initial programming phase, 77.8% (7/9) patients showed a positive response within hours to days, manifested as amelioration of motor symptoms, relaxation of neck muscles, or relief of pain. When the symptom improvement got the stable phase, patient-1 reduced amplitude from 2.0 to 1.0 V for two years and patient-2 reduce amplitude from 3.5 to 2.5 V for half a year, all of them maintained a steady improvement as before.

### Adverse events

There were no serious adverse events related to the surgical procedure (for example: intracranial hemorrhages or device infections) in all patients during the follow-up. The uncomfortable tensor sensation near the extension wire was experienced in 4 patients caused by the extension wire, but no patient needed additional surgery. One patient experienced mild pain sometimes at the neurostimulator site, but it had no practical impact on her daily living. One patient experienced mild balance disturbances, two patients experienced mild hand weakness and eight patients experienced dyskinesia, all of them were alleviated by programming.

## Discussion

The present study established the safety, efficacy, and reliability of STN DBS for the treatment of refractory isolated CD. The results showed that patients attained persisting and substantial improvement of various dystonic manifestations and the quality of life.

Unlike other study reported^11^, we did not observed obvious micro-lesions effect just like Parkinson's disease after DBS. The improvement on the TWSTRS total score was 22.2% ± 11.7% at 1 month after STN-DBS for CD, which was lower than the improvement for primary dystonia reported in other investigations (more than 50%)^13,14^. The lower improvement degree may be related to two reasons. The first, we usually used lower amplitude (1.5–2.0 V) in the first month after neurostimulator initiated for avoided the side effects, which may not get the ideal treatment threshold. The second, CD only involves cervical symptoms, which is different from segmental, multifocal or generalized dystonia involving multiple parts of the body. As the increase of amplitude, the improvement reached 55.3% ± 20.3% by the 3-month visit, similar to that previously reported in the follow-up study of primary CD patients treated with STN DBS (50.6%)^12^. To our surprise, after 6 and 12 months of STN stimulation, the improvement reached 74.3% ± 29.8% and 87.2% ± 27.0%, respectively, which were superior to that previously report showed an improvement of 60.2% and 62.9% at the corresponding point in time^12^.

The long-term improvement was more remarkable and superior to previously study of idiopathic predominantly CD treated with STN DBS^15^. A study of STN DBS in the treatment of primary dystonia also showed similar surprising results, with an average improvement rate of 85.0% in BFMDRS-M at 1 year, 90.8% at 5 years and 91.4% at the last follow-up^14^. Ultimately, the quality of life in all patients improved significantly, as reflected in the SF-36 assessment. No matters in physical or mental aspects, dystonia symptoms were the main reason for the decline of quality of life. Therefore, with the significant improvement of dystonia symptoms after STN DBS, the patients get rid of the trouble and disgrace of the disease, resume normal daily activities and social participation, and enhance their confidence in life.

The target choice of GPi or STN is still controversial in treatment of dystonia and the former is more popular up to now. Although existing control study and meta-analysis shown no statistical difference between the two targets in the treatment of primary dystonia^10,16^, however, the reliability of this conclusion was questionable due to the shorter follow-up time, limited sample size and different subtypes of dystonia. Actually, convergent evidence now suggests that CD is a ‘network disorder’ resulting from dysfunction in multiple different brain regions, and the abnormal connectivity between cerebellar and somatosensory has been confirmed^17^. Current studies have found that STN not only regulates the excitatory output of GPi through indirect and hyperdirect pathways^18^, but also plays an important role in the functional connectivity in the cortico-basal ganglia-cerebellar network^19^. Therefore, STN-DBS may have certain advantages in the treatment of CD. Obviously, our results showed that the effects of STN DBS seemed to be dramatically superior to those of GPi DBS for isolated CD^8,20^. The trend to maintain the symptoms improvement continues at one year and beyond after STN DBS is incomparable for GPi^20^. The response after STN stimulation is immediate in most patients, which can provide clues for selecting the optimal stimulus contact and parameters. The same phenomenon was observed by other authors^11,14^. This advantage over GPi-DBS is conducive to improving the efficiency and quality of programming, making patients full of confidence and obtaining satisfactory experience. In present study, the parameters of stimulation were significantly lower than those of GPi stimulation^20,21^. Therefore, another advantage of STN over GPi is longer battery life.

Previous studies on the treatment of primary CD with GPi DBS have reported that some of them present bradykinesia and axial motor symptoms^22,23^, and dysarthria is another most common stimulation-induced side effect with a prevalence estimated up to 12%^24^. In general, these problems cannot be solved through programming changes without sacrificing dystonia improvement. By comparison, no patients reported slowing of motor function and dysarthria in the present series. Dyskinesia is a temporary side effect and easily induced by voltage-limiting stimulation, which typically improved by programming changes without losing clinical benefit. Dyskinesia can usually be minimized or avoided by decelerate the rate at which the amplitude was increased in the STN or activating the contact located more dorsal STN. Although no side effects such as dysarthria, dysphoria, anxiety, depression, dysphasia, numbness, paresthesia, or pain occurred in our study, they have been reported previously^15^. This may be attributable to the smaller size and topological organization of the STN, without well-defined anatomical boundaries and instead a degree of overlap between functional zones^25^. This means that stimulation can more easily affect internal non-motor functional areas and external surrounding tissues. Therefore, accurate electrode position, ideal active contact selection and reasonable programming are especially important.

In our experience, the ideal position for electrode implantation is the central of the dorsal STN and the optimal stimulation points is the area above the STN (including the Zi and Forel H2), which is similar to treating Parkinson's disease and confirmed again by a study using directional leads^26^. We found that patient’s self-adjustment under the supervision of the treating physician is an advanced DBS programming strategy, feasible, practical and significantly shorten consultation time in dystonia patients. It was not easy to supervise patients who were far away from treatment center, so that they could not reprogram in time, then affected the treatment effectiveness. Fortunately, the modified DBS system which can offer video communication and remote programming function allowing doctors to learn about the treatment effect timely, select optimal active contacts and adjust appropriate stimulation parameters for patients and significantly promote the efficiency of programming.

Patient-5, an unusual case, whose sustained cervical dystonia symptom before DBS changed to paroxysmal episodes under constant-voltage stimulation after DBS. The unexpected thing was that paroxysmal dystonia symptom disappeared immediately after converted constant-voltage stimulation to constant-current stimulation without a change in active contact. It seems that constant-current stimulation can relieve paroxysmal dystonia symptom, which may be related to its ability to offer more stable stimulation^27^. This was just an isolated case and further attempts are necessary.

In order to alleviate or avoid stimulus-related side effects, the “Top-down” rule is adopted in the treatment of dystonia with Gpi-DBS^28^. We adopted a similar programming strategy for individual patients,, called “Up-top-down” rule, which aims not only to alleviate or avoid stimulus-related side effects, but also to save stimulator energy consumption (e.g. patient-1 and 2). Some studies reported that cessation of STN stimulation does not aggravate dystonia^14,29,30^, but our patient-7 turned off the stimulator inadvertently leaded to partly relapse and then returned to normal when turned on. Therefore, we suggest that a modest reduction of the stimulation amplitude when get the stable phase can save stimulator power, reduce the side effects of stimulation and maintain the therapeutic effect, but not recommended to turn off the stimulator or reduce the amplitude when the symptom improvement is not reach the stable phase.

The predictive factors associated with outcome of DBS are also one of the hottest topics in discussion. Some long term outcome studies of STN and dystonia have shown that the degree of improvement is related to duration of the disease before surgery^13^ and age at surgery^15^. In present series, the long-term outcome seemed to be not associated with age at disease onset, disease duration, sex, type of dystonic movement, age at surgery, duration of stimulation, and baseline TWSTRS severity.

The primary device-related adverse event in our study was uncomfortable tensor sensation near the extension wire, which has been reported^31^. The extension wire after DBS even trigger painful CD, deeper channeling of the wire extensions produced a complete remission^32^. Discomfort around neurostimulator is another reported device-related adverse event, which related to the size of neurostimulator^15^. Future advances in materials and technological improvement may allow patients have a better treatment experience.

Several limitations were existed in present study. First, this is a single-center non-blinded retrospective study and the results are limited to our relatively small sample size and patient selection. Although all patients had prominent cervical involvement, three patients also had other body regions affected. Thus, large controlled multicenter double-blinded are needed to identify whether the observed strong therapeutic effects of STN-DBS can be confirmed in other research settings, in other samples of patients with refractory isolated CD. Furthermore, none of the patients underwent genetic testing. CD has a complex genetic background^33^, with various underlying causes. Identification of genetic variations associated with CD may conducive to understand the association with treatment.

## Conclusions

The present retrospective study found that the vast majority of patients with refractory isolated CD can obtain enormous benefits in the aspect of reduction in dystonic symptoms and improvement in quality of life after bilateral STN DBS. The improvement of individual patient has a tortuous process. Therefore, large controlled multicenter double-blinded trials are warranted to identify these observations on account of the small sample size of the current study. Most importantly, the therapy expatiated here seems to be a safety, effective and stable method, but further follow-up is required.

## Methods

### Subjects

This study was performed at the Sixth Medical Center of Chinese PLA General Hospital. All of the patients and their family assent to this trial and signed the informed consent form. Between September 2014 and June 2018, nine consecutive patients with refractory isolated CD who had undergone bilateral STN DBS at our center were eligible to participate in the study. Inclusion criteria were: a diagnosis of isolated predominantly CD; severe functional impairment and failed to respond to oral pharmacotherapy, botulinum toxin or selective peripheral denervation (SPD); absence of secondary causes including neuroleptic treatment before dystonia onset; normal neurologic examination except for dystonia and normal brain MRI; and willing to accept regular consulting visits and a long-term follow up. Exclusion criteria were: a medical contraindication to surgery; evidence on MRI of another neurologic disorder or extensive brain atrophy or anatomical anomalies in the basal ganglia region; severe cognitive impairment or depression, severe psychiatric disease. The study was conducted in accordance with the 1964 Helsinki Declaration (2013 revision) and it was approved by Ethics committee of the Sixth Medical Center of Chinese PLA General Hospital.

### Surgical procedures

**Figure 2:**
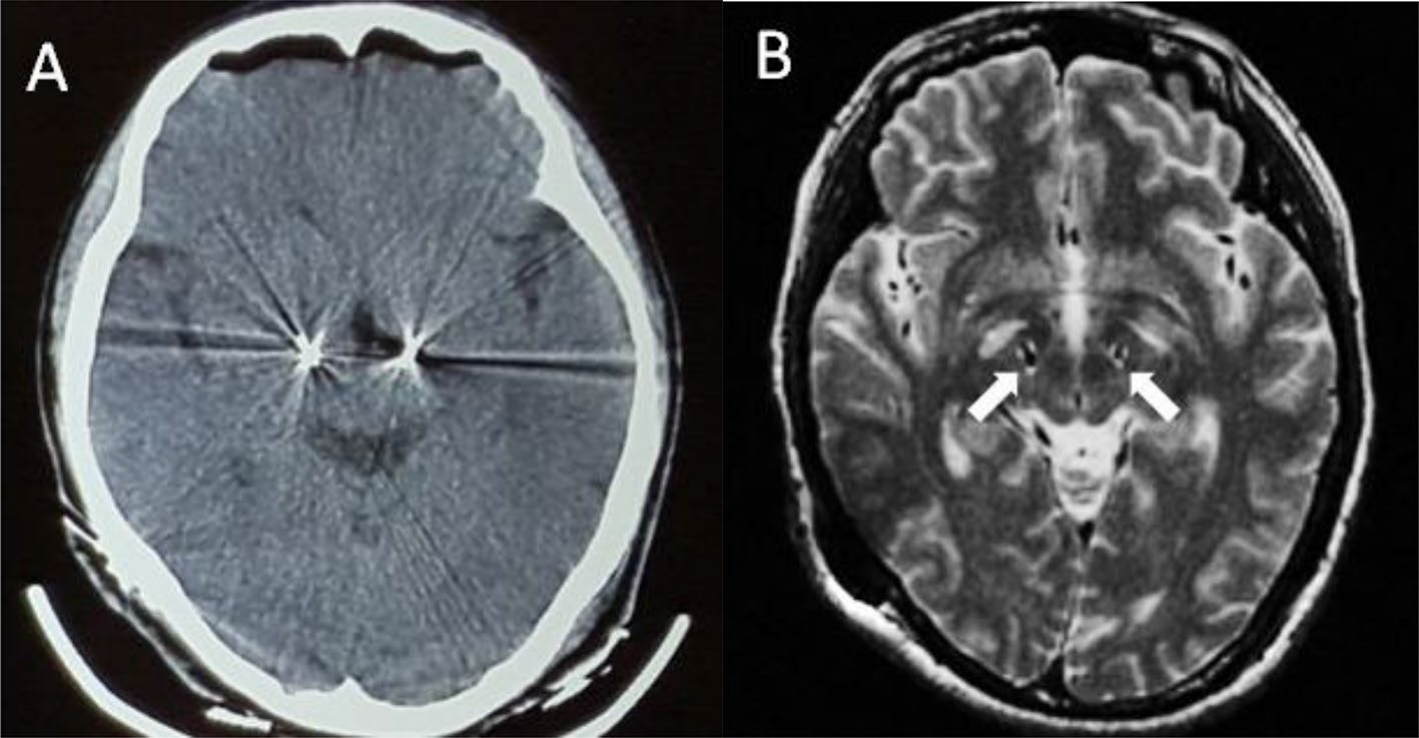
Case 4. Postoperative brain CT (**A**) for ruling out haemorrhage and axial T2-weighted MR images (**B**) for showing the targets location (white arrow) of the subthalamic nucleus.

All operations were accomplished by the same two skillful neurosurgeons and the surgical technique was similar to previously described^34^. The dorsal regions of bilateral STN were chosen as the targets which were determined directly by T2 MRI or by fusing 3 T MRI (T1/T2 sequences) obtained a day before the surgery with high-resolution CT (scanned with a Leksell stereotactic frame). Quadripolar DBS leads, which had four contacts (contact width of 1.5 mm, diameter of 1.3 mm and the gap between two adjacent contacts is 0.5 mm), were implanted guided by microelectrode recording under local anesthesia, and then connected to a dual channel neurostimulator implanted in the right subclavicular pocket under general anaesthesia (devices model see Table 1). All patients experienced post-operative brain CT scan to rule out haemorrhage (Fig. 2a). Post-operative electrodes positions were identified by MRI scan (Fig. 2b) or by fusing post-operative high-resolution CT images with the pre-operative MRI.

### Post-operative programming

Programming was initiated 3 weeks after DBS surgery. Each contact was activated in monopolar stimulation and constant-voltage stimulus mode respectively, with pulse width, frequency and amplitude of 60 us, 130 Hz and 1.5–2 V, respectively. The treatment responses and side effects were observed and recorded. The contact chosen at initial programming was the one clinical improvement relatively significant and would allow for the increase amplitude in increments of 0.05–0.1 V per week without stimulation-induced adverse events (with the exception of dyskinesia). Initial amplitude set between 1.0 and 1.5 V and then slowly increased by the patient in the following time to further treat the dystonia. If dyskinesia occurred, patients were instructed to slightly decrease the amplitude and then slowly increased. If symptoms improvement was not desirable at amplitude of 3.0 V or stimulation-induced adverse events limited the increase in amplitude, the patient was reprogrammed by doctor using an additional dorsal contact, even bipolar stimulation or constant-current stimulus mode were tried.

### Clinical evaluation

Dystonic symptoms were evaluated by an independent movement disorder neurologist at pre-operation [baseline] and post-operation [short-term follow-up: 1, 3, 6 and 12 months after neurostimulator turned on; long-term follow-up: 24 months and last follow-up], according to the Toronto Western Spasmodic Torticollis Rating Scale (TWSTRS)^35^, which contains three sections, a severity score (0–35), a disability score (0–30), and a pain score (0–20). Higher scores represent greater impairment. The 36-item Short-Form General Health Survey (SF-36)^36^ was used to evaluate their health-related quality of life at baseline and the last follow-up, which includes eight aspects of the health condition of each patient, with the score for each section ranging from 0 (worst) to 100 (best), and higher scores indicate better daily function and condition.

### Statistical methods

SPSS version 19.0 (SPSS, Inc., Chicago, IL, USA) was used for statistical analyses. The Shapiro–Wilk test was applied to analyze the distribution of the grouped data. The two tailed paired-sample *t* tests was used to compare baseline and follow-up TWSTRS scores (severity, disability, pain, and total) and SF-36 scores if the data were distributed normally. If not, the Wilcoxon matched-pairs signed-rank test was performed. Correlations between the final improvement in the TWSTRS severity subscores and the relevant variables were analyzed by using Spearman correlation (for quantitative variables) or the Mann–Whitney test (for categorical variables). Two-tailed p < 0.05 were considered statistically significant. The results are presented as mean ± SD.

### Ethical approval

Ethics approval/consent to participate/consent for publication.

### Informed consent

All patients gave their written informed consent to surgery, the study and the publication of results.
